# Cloning and expression heterologous alanine dehydrogenase genes: Investigation of reductive amination potential of L-alanine dehydrogenases for green synthesis of alanine derivatives

**DOI:** 10.1016/j.heliyon.2024.e26899

**Published:** 2024-02-29

**Authors:** Ğarip Demir, Jarkko Valjakka, Ossi Turunen, Fatih Aktaş, Barış Binay

**Affiliations:** a Department of Molecular Biology and Genetics, Gebze Technical University, 41400, Gebze, Kocaeli, Turkey; b Faculty of Medicine and Health Technology, Tampere University, FI-33100, Tampere, Finland; c School of Forest Sciences, University of Eastern Finland, FI-80101, Joensuu, Finland; d Faculty of Engineering, Düzce University, 81600, Düzce, Turkey; e Department of Bioengineering, Gebze Technical University, 41400, Gebze, Kocaeli, Turkey; f BAUZYME Biotechnology Co., Gebze Technical University Technopark, 41400, Gebze, Kocaeli, Turkey

**Keywords:** Unnatural amino acids, L-Alanine dehydrogenase, Reductive amination mechanism, Active site modelling, Green synthesis

## Abstract

Unnatural amino acids (UAAs) offer significant promise in a wide range of applications, including drug discovery, the custom design of peptides and proteins, and their utility and use as markers for monitoring molecular interactions in biological research. The synthesis of UAAs presents a formidable challenge and can be classified into two primary categories: enzymatic and chemical synthesis. Notably, the enzymatic route, specifically asymmetric synthesis, emerges as a an attractive method for procuring enantiopure UAAs with high efficiency, owing to its streamlined and concise reaction mechanism.

The current study investigated the reductive amination activity mechanisms of alanine dehydrogenase (L-AlaDH), sourced from a combination of newly and previously characterized microorganisms. Our principal aim was to evaluate the catalytic efficiency of these L-AlaDH enzymes concerning a range of specific ketoacids and pyruvate to ascertain their capability for facilitating the production of both natural and unnatural amino acids. After the characterization processes, mutation points for *Tt*AlaDH were determined and as a result of the mutations, mutants that could use ketocaproate and ketovalerate more effectively than the wild type were obtained.

Among the enzymes studied, *Met*AlaDH exhibited the highest specific activity against pyruvate, 173 U/mg, and a K_M_ value of 1.3 mM. *Vl*AlaDH displayed the most favourable catalytic efficiency with a rate constant of 170 s^−1^mM^−1^. On the other hand, *Af*AlaDH demonstrated the highest catalytic efficiency against α-ketobutyrate (34.0 s^−1^mM^−1^) and α-ketovalerate (2.7 s^−1^mM^−1^). Of the enzymes investigated in the study, *Tt*AlaDH exhibited the highest effectiveness among bacterial enzymes in catalyzing ketocaproate with a measured catalytic efficiency of about 0.6 s^−1^mM^−1^ and a K_M_ value of approximately 0.3 mM. These findings provide valuable insights into the substrate specificity and catalytic performance of L-AlaDHs, enhancing our understanding of their potential applications in various biocatalytic processes.

## Nomenclatures

l-alanine dehydrogenase: (L-AlaDH)
Unnatural amino acids: (UAA)
Multi-enzymatic cascade: (MEC)

## 1 Introduction

The standard genetic code encodes for 20 proteinogenic amino acids. However, researchers have identified a range of non-proteinogenic amino acids that occur naturally in living systems, expanding the number of naturally occurring amino acids beyond the 20 specified by the genetic code. To date, more than 800 noncoded amino acids have been identified, each possessing unique chemical properties and biochemical roles [1,2]. In addition to these naturally occurring amino acids, there are also numerous synthetic unnatural amino acids (UAA) that have been synthesised in laboratory settings, but are not found in nature [1,2]. The two primary categories of UAA are distinguished by their structural similarity to amino acids (AA). If an UAA has a similar structure to AA, it is referred to as an "analogue", while UAA that differ significantly from typical AA structures are known as "surrogates" [2,3]. These molecules play essential roles in the biochemistry, food, and pharmaceutical industries, contributing to the development of novel drugs, nutritional supplements, and other valuable products [2].

Despite their enormous potential and their ability to be used in different fields, the asymmetric synthesis of UAA remains a challenge. Asymmetric synthesis of UAA can be grouped under two main headings: Enzymatic (green) and chemical synthesis. Chemically synthesised UAA can be achieved through various chemical reactions, such as alkylation [2], cyclisation [4], arylation [5], acylation [3] and side chain modification [6]. Although there are different methods for chemical synthesis of UAA, the chemicals used in the synthesis are harmful to the environment, the conditions used for synthesis are difficult, and the inability to synthesise enantiopure UAA are the main problems encountered in chemical synthesis [[2], [3], [4],^6^]. The production of UAA through chemical synthesis typically entails the application of racemic purification techniques. The utilisation of such methods may necessitate the use of chemical agents that can be hazardous to human health and the environment. In addition, the separation of enantiomers required for the production of desired enantiomers includes the high financial cost of chiral separation [2]. Applications of UAA are multifarious, encompassing various fields of scientific research. Their potential use ranges from the creation of novel drug compounds [7] to acting as probes in experimental investigations, the development of peptides and proteins with unique properties or functionalities [8,9], serving as markers or labels in biological research and their use in tracking specific molecules or cellular interactions [[9], [10], [11]].

The enzymatic route for synthesis of chiral enantiopure UAA is an appealing approach due to the efficient and concise reaction process. This method often uses optimal buffers as the reaction medium. The use of enzymes eliminates the need for additional protective groups and makes the process a more energy-efficient, sustainable, and cost-effective technology for the production of valuable UAA. Asymmetric UAAs are frequently produced using ammonia-lyases [12] transaminases [13,14] and l-amino acid dehydrogenase [15,16], Additionally, the multi-enzymatic cascade (MEC), another biocatalytic technique, is employed for UAA synthesis [17]. Also, various enzymes, including acylases, lipases, nitrilases, and hydantoinase [18], have been utilized in the synthesis of a diverse array of chiral amino acids through kinetic resolution [1]. Apart from these designed enzymes with novel capabilities offers a versatile platform for biocatalytic hydroamination [19] l-alanine dehydrogenase (L-AlaDH) has been shown to be a promising and innovative approach to the synthesis of UAA through its reductive amination mechanism. Moreover, the use of enzymes, such as L-AlaDH, can facilitate the production of enantiomerical pure UAA, which is crucial for their use in biochemical and pharmaceutical applications [8,9]. Among chiral organic compounds, alanine and its derivatives have great potential for use as precursors because of their small chiral structure [20].

**Fig. 1:**
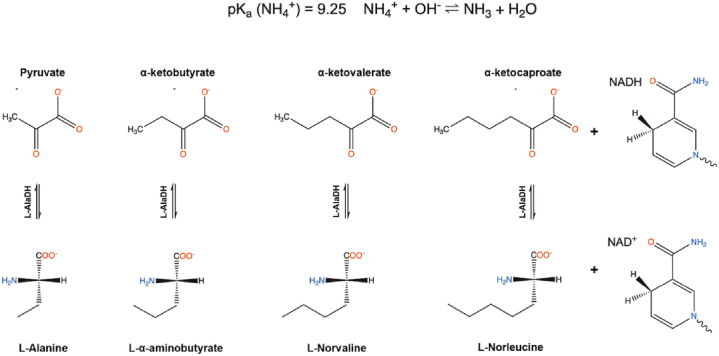
Schematic description of the molecular structures of the catalytic reactions with l-alanine dehydrogenase (L-AlaDH). Ketoacids act as substrates with NADH, and l-amino acids with NAD ^+^ are products in the reducing amination reaction. The amount of ammonia required in the amination reaction is dependent on the alkalinity of the buffer solution and the pK_a_ value of the ammonium ion.

Many preferred asymmetric methods for UAA synthesis involve reductive amination reactions, although it is evident from previous studies that the reductive amination kinetic values of L-AlaDH are quite limited [21] Therefore, a study of new enzyme variants could highlight pathways to improve reaction efficiency.

l-alanine dehydrogenase (L-AlaDH) (EC 1.4.1.1) belongs to the amino acid dehydrogenase group in the family of oxidoreductases. The enzyme is NAD(H) dependent and reversibly converts l-alanine to pyruvate. The reversible reductive amination reaction, catalysed by L-AlaDH, results in the synthesis of l-alanine and its derivatives from pyruvate and keto acids, respectively (Fig. 1).

Research on the reductive amination mechanism of L-AlaDHs has been conducted in various organisms, including *Streptomyces coelicolor* [22], *Archaeoglobus fulgidus* [23], *Vibrio proteolyticus* [24], *Phormidium lapideum* [25] and *Helicobacter aurati* [26]. However, a comprehensive understanding of the factors that underpin the reaction mechanism remains elusive. These factors may include either a specific acid-base reaction or a hydride transfer reaction, and depend on the AA at the active site and all the solutes in aqueous solution [[27], [28], [29], [30], [31]]. To gather crucial data on the reductive amination activity of L-AlaDHs, we selected six enzymes from diverse sources and performed comprehensive characterization of their reductive activity, while also using molecular modeling to study enzyme functioning. Through utilisation of these methods, we aimed to gain a better understanding of the reductive amination mechanism of L-AlaDHs and their potential application in the synthesis of UAA.

Here, for the first time, we report the heterologously expressed L-AlaDHs from *Melghiribacillus thermohalophilus* (*Met*), *Ammoniphilus* sp. CFH 90114 (*Acfh*), *Vagococcus lutrae* (*Vl*), *Candidatus Bathyarchaeota archaeon* (*Cb*), and the previously studied *Thermus thermophilus* (*Tt*) and *Archaeoglobus fulgidus* (*Af*) in *E. coli*. The reductive amination activity of L-AlaDHs was investigated with regard to pyruvate and its derivatives (α-ketobutyrate, α-ketovalerate and α-ketocaproate).

## 2 Materials and methods

Unless otherwise stated, all chemicals utilized in the experiments were bought from Sigma-Aldrich (Missouri, USA). The *E. coli* BL21 (DE3) cells used in enzyme expression were purchased from Invitrogen (Massachusetts, USA). Chemicals purchased from LABM (Bury, UK) were used to create all culture media. The kit used in plasmid puriﬁcation was purchased from Invitrogen, the Ni-NTA HisTrap column was purchased from General Electric (Boston, MA, USA), and the PD-10 columns were purchased from MilliporeSigma (Burlington, MA, USA).

### 2.1 Cloning of the *alds* genes into the pET28a (+) vector

Alanine dehydrogenase genes from *Melghiribacillus thermohalophilus* (UniProt accession number: A0A4R3N762)*, Thermus thermophilus* (UniProt accession number *Q5SLS7**), Archaeoglobus fulgidus* (UniProt accession number: O28608), *Candidatus Bathyarchaeota archaeon* (UniProt accession number: A0A7J3W6F5), *Vagococcus lutrae* (UniProt accession number: A0A429ZGT5) and *Ammoniphilus* sp. CFH 90114 (UniProt accession number: A0A4Q1SWL0) coding for L-AlaDH enzymes, were optimised by codon usage for *E. coli* BL21 (DE3) expression. Optimised gene sequences were purchased from Gene Universal (Delaware, USA). The synthesised L-AlaDH genes were ligated into the pET-28a(+) plasmid and transformed into competent NEB® *E. Coli* DH5α cells. The recombinant plasmid pET28a(+) was used to express *ald* coding region introduced between the *Nde*I and *Xho*I restriction sites and including the 6xHis-tag at the *N* -and *C*-terminal ends.

### 2.2 Protein expression and enzyme purification of L-AlaDH

*E. coli* BL21 (DE3) cells were used as the host to express L-AlaDH from the pET-28a(+) plasmid. Vectors that contained *ald* genes were transformed into host cells via the heat-shock transformation protocol from NEB®. Individual colonies from each LB (Luria-Bertani)-agar plate that was supplemented with 50 μg/mL kanamycin were selected, inoculated onto 10 mL LB medium that contained kanamycin (50 μg/mL), and then incubated at 37 °C at 200 rpm overnight. To heterologously express the proteins, the overnight culture was transferred to 100 mL Studier medium that contained 50 μg/mL kanamycin and was then incubated overnight at 30 °C at 200 rpm [32]. After 16 h of incubation, the cells were collected by centrifugation at 4.500 rpm for 15 min (at 4 °C).

Cell pellets were resuspended in 10 mM NaPi buffer (pH 7.4) that contained 1 mg/mL lysozyme and 0.5 mM PMSF, and were then incubated on ice for 30 min. The resuspended cells were disrupted by sonication and clarified by centrifugation (11,000 rpm for 45 min). Cell lysates were purified by Ni^2+^ affinity chromatography as follows: After the crude enzyme mixture was loaded onto the column, the column was washed with 5 mL of Buffer A, which contained 30 mM of imidazole, and then the L-AlaDHs were eluted with buffers that contained different imidazole concentrations (100, 200, 400, and 500 mM imidazole). Cell lysates were analysed by SDS-PAGE in order to identify the expressed proteins [33].

### 2.3 Determination effect of pH and temperature on L-AlaDH enzyme activity

Purified L-AlaDH enzyme activity was measured at pH 4–11: 50 mM sodium citrate for pH levels 4 to 6; 50 mM Tris-HCl for pH levels 7–9; 50 mM sodium carbonate and glycine for pH levels 9.5–10.5, sodium bicarbonate for pH 11. For reductive amination of pyruvate, the reaction was monitored by following the consumption of NADH at 340 nm. To determine the optimum temperature for L-AlaDH activity, the reductive amination reaction for bacterial L-AlaDHs was tested at temperatures between 25 and 70 °C, while archaeal L-AlaDHs were tested at temperatures between 25 and 90 °C. Each enzyme was studied at its optimal pH.

### 2.4 Determination of kinetic parameters

To determine the kinetic parameters of the purified L-AlaDH enzymes, multiple measurements were conducted using pyruvate and its derivatives (α-ketobutyrate, α-ketovalerate and α-ketocaproate) at different concentrations. Specifically, pyruvate concentrations ranged from 0.1 to 10 mM, while derivative concentrations ranged from 0.1 to 50 mM. These tests were performed under optimal pH and buffer conditions with 0.5 M NH_4_Cl. The reaction was initiated by adding 0.5 mM NADH and pure L-AlaDH enzyme. Specific activity was calculated as units/mg using ε = 6220 M^−1^ cm^−1^ and by monitoring the decrease in absorbance based on NADH depletion over 10 min at 340 nm. One unit is defined as the amount of enzyme required for the formation of 1 μmol of NAD^+^ in 1 min [34]. The first-order rate constant k_cat_ and Michaelis constant K_M_ values of the purified enzymes were determined by drawing Michaelis-Menten graphs using the given activity values and utilizing GraphPad Prism 9 (GraphPad Software, San Diego, USA). Moreover, the conversion efficiency of the L-AlaDHs over ketoacids was computed based on NADH consumption per unit time, following the approach employed by Dedeakayoğulları et al. [35]**.**

### 2.5 Molecular modelling

Multiple Sequence Alignment of *T. thermophilus* (*Tt*AlaDH), *Ammoniphilus* sp*. CFH90114* (*Acfh*AlaDH), *M. thermohalophilus* (*Met*AlaDH), *V. lutrae* (*Vl*AlaDH), *A. fulgidus* (*Af*AlaDH) and *C.B. archaeon* (*Cb*AlaDH) was carried out with EMBL-EBI Clustal Omega Server. The protein data bank contained the PDB 2EEZ 3-D structure [36] for *T. thermophilus* without substrates and cofactors, and the 1omo 3-D structure for *Archaeoglobus fulgidus* (*Af*) was also available but only with a cofactor [28]. Since PDB 2EEZ and PDB 1OMO exist as open conformation, which are non-reactive structures in the protein data bank, their structures were modelled to be in a closed reactive conformation using PDB 2VOJ (reactive closed conformation structure of *Mycobacterium tuberculosis*) as a template [29] The modelled protein segments with the substrate and NADH in PDB 2EEZ were superimposed on the PDB 2VOJ structure by changing the positioning of the PDB 2EEZ segments to match the PDB 2VOJ structure. The "missing" loops of the PDB 2EEZ model were built by the Swiss-PdbViewer program to be similar to the PDB 2VOJ [34] and then the geometry of the model structure was fine-tuned with the energy minimisation program of the Yasara minimisation [29,37,37,38].

## 3 Results

### 3.1 Expression and puriﬁcation of AlaDH

The *ald* genes from *M. thermohalophilus* (*Met*), *T. thermophilus* (*Tt*), *A. fulgidus* (*Af*)*, C.B..archaeon* (Cb)*, Vagococcus lutrae* (Vl)*, Ammoniphilus* sp. *CFH 90114* (*Acfh*) were cloned into the expression vector pET-28a(+). The plasmids that contained the *ald* genes were transformed into *E. coli* BL21 (DE3) cells with *N*- and *C*-terminal 6xHis-tags. Following purification, the purity of the eluted enzyme samples was evaluated by SDS-PAGE analysis. All L-AlaDH enzymes were successfully expressed in *E. coli*, as evidenced by the presence of their respective protein gel bands (Fig. S1). Moreover, the observed molecular weight of the subunits was in agreement with the calculated molecular weight based on the protein sequence (Fig. S1).

### 3.2 Effect of pH and temperature on L*-*AlaDH activity

**Fig. 2:**
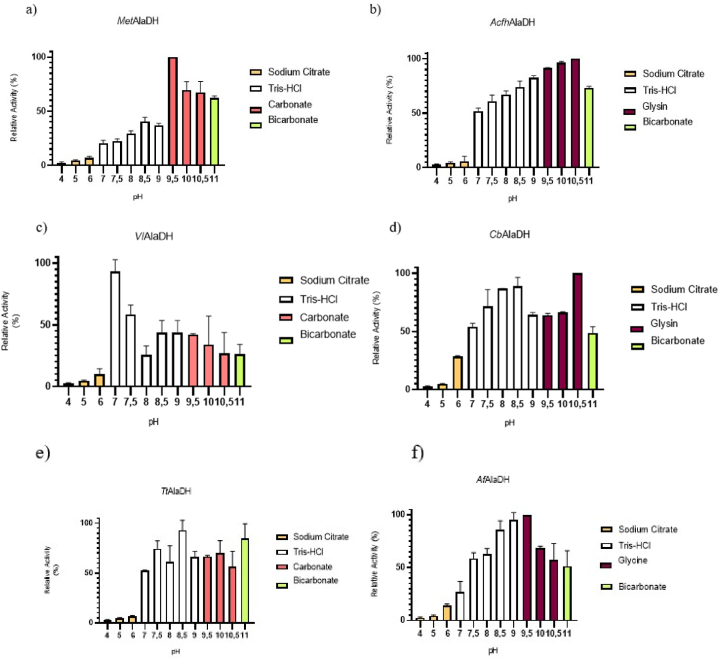
Effect of pH on the activity of L-AlaDH enzymes in reductive amination reactions (a) *Met*AlaDH, (b) *Acfh*AlaDH, (c) *Vl*AlaDH, (d) *Cb*AlaDH, (e) *Tt*AlaDH, (f) *Af*AlaDH. L-AlaDHs activities were measured at 25 °C in different buffer systems.

Both pH (4–11) and temperature (25–90 °C) affected L-AlaDH activity when pyruvate was used as the substrate. The optimal pH values for reductive amination were determined at 25 °C for *Acfh*AlaDH, *Af*AlaDH, *Cb*AlaDH, *Met*AlaDH, *Vl*AlaDH and *Tt*AlaDH enzymes (Fig. 2). Typical to this group of L-AlaDH enzymes was that the pH profile varied considerably, and that the enzyme was mostly active above pH 7 (Fig. 2(a–f)), while only minor activity was observed below pH 7, and the relatively highest pH 6 activity was shown by *Cb*AlaDH (Fig. 2 (d**)**). Only one enzyme showed an activity peak around neutral pH values, with the highest activity observed at pH 7 for *Vl*AlaDH (Fig. 2 (c**)**) in the Tris buffer. All other enzymes showed activity peaks between pH 8.5–10.5: *Tt*AlaDH at pH 8.5 in the Tris buffer, *Af*AlaDH and *Met*AlaDH at pH 9.5 in the glycine and carbonate buffer, and *Acfh*AlaDH at pH 10.5 in the glycine buffer (Fig. 2(a–f)). *Cb*AlaDH showed the highest activity in the glycine buffer at pH 10.5 (Fig. 2(d). The variable pH graphs and activation effects indicated that the L-AlaDH enzymes were quite sensitive to very specific electrodynamic effects.

The high pKa value for ammonium (9.25) was compatible with the high pH optimum value of the L-AlaDH enzymes, which indicated that enzymatic activity increased considerably in tandem with increased alkalinity, in which case the amount of ammonia is higher according to its formation reaction NaOH + NH_4_Cl ⇌ NH_3_ + H_2_O + NaCl.

The alkaline environment can also affect the mechanism of the enzyme as Grimshaw and Cleland have shown in their study [31] that when pH is at pH 9.35, and levels of ammonia are above 50 mM it may cause uncompetitive substrate inhibition, which may also occur in the reactions with two or more substrates or products [31].

**Fig. 3:**
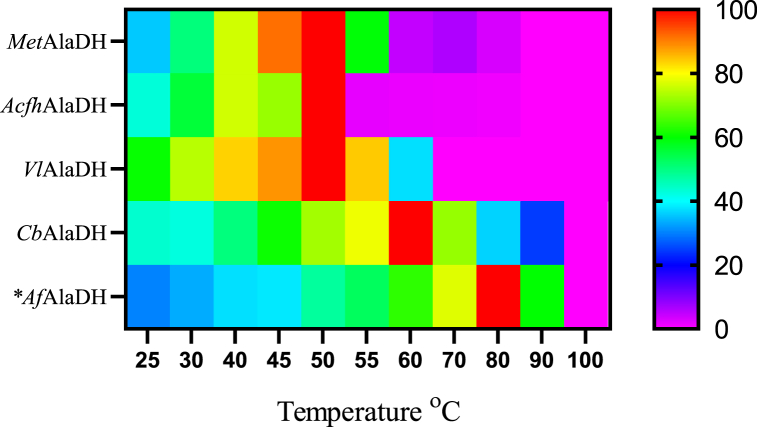
Effect of temperature on the activity of L-AlaDH enzymes in reductive amination reactions. *Data taken from the literature [23].

The effect of temperature on the reductive amination activity of L-AlaDH varied considerably between the studied enzymes. With the exception of CbAlaDH (optimum at 60 °C), all the enzymes characterized in this study showed highest activity at 50 °C (Fig. 3), although *Acfh*AlaDH rapidly lost activity at temperatures above this value. While *Met*AlaDH activity almost ceased at 60 °C, *Vl*AlaDH had the highest temperature tolerance among the bacterial L-AlaDHs and activity only ceased at 70 °C. In our study, *Af*AlaDH was the only example of an L-AlaDH enzyme derived from an archaeal source with well-established characteristics. This enzyme exhibited its highest level of activity at a temperature of 82 °C [23]. *Cb*AlaDH, another enzyme that originates from archaeal sources, exhibited its highest activity at 60 °C. Of the enzymes in our study, *Cb*AlaDH showed the highest temperature tolerance, being active up to 90 °C. It should also be noted, however, that the solubility of ammonia decreases as temperature rises, thus lowering the concentration of ammonia in the reaction when a constant concentration of ammonium ions was used in the assays. This behaviour probably lowers the apparent temperature optimum of L-AlaDH enzymes to some degree.

### 3.3 Kinetic parameters

The analysis of specific activity values presented in (Table 1) indicated that all examined L-AlaDH enzymes were active in pyruvate, α-ketobutyrate and α-ketovalerate substrates. Notably, *Met*AlaDH, exhibited the highest specific activity with regard to pyruvate and *Cb*AlaDH exhibited the worst specific activity against pyruvate (Table 1), and all L-AlaDH enzymes exhibited significantly higher activity with regard to pyruvate than the other substrates. In addition, α-ketobutyrate was found to be the preferred substrate after pyruvate among the tested L-AlaDH enzymes. It should be noted that only the *Af*AlaDH and *Tt*AlaDH demonstrated activity with regard to α-ketocaproate, while the other L-AlaDH candidates did not exhibit any activity with regard to this substrate under the studied conditions. These findings clearly showed that the carbon backbone length of the substrates had a strong effect on the ability of the enzyme to use it as a substrate. It was also found that enzymes from different sources varied in this respect.

**Table 1:** Optimum pH and specific activity values (U/mg) of purified L-AlaDH enzymes with regard to different substrates.

Enzyme	pH	Pyruvate	α-ketobutyrate	α-ketovalerate	α-ketocaproate
***Met*AlaDH**	9.5	172.5 ± 5.0	3.7 ± 0.4	0.9 ± 0.1	ND^a^
***Acfh*AlaDH**	10.5	154.2 ± 8.0	6.7 ± 0.3	1.2 ± 0.1	ND^a^
***Vl*AlaDH**	7.0	134.8 ± 3.0	13.2 ± 0.5	1.9 ± 0.1	ND^a^
***Tt*AlaDH**	8.5	127.0 ± 3.0	1.7 ± 0.3	1.5 ± 0.0	0.3 ± 0.0
***Cb*AlaDH**	10.5	3.2 ± 0.3	1.6 ± 0.2	0.4 ± 0.0	ND^a^
***Af*AlaDH**	9.5	114.2 ± 6.0	4.3 ± 0.3	0.5 ± 0.1	2.8 ± 0.3

a None determined.

Michaelis-Menten graphs were created by assaying the L-AlaDH enzymes for each substrate at 25 °C and an optimum pH level to determine the kinetic parameters of the enzyme. GraphPad Prism 9 (GraphPad Software, 2022) was used to determine the kinetic parameters (k_cat_ and K_M_) and the summary of the kinetic parameters obtained for pyruvate (Table 2) revealed significant diversity in both K_M_ and k_cat_ values. The K_M_ values for the studied enzymes fell within the range 0.2–1.3 mM, while the range of k_cat_ values was 0.5–139.6 s^−1^ mM^−1^. Notably, *Met*AlaDH exhibited the highest k_cat_ value among the tested enzymes, and also had the highest K_M_ value. *Vl*AlaDH exhibited the highest catalytic efficiency value of the L-AlaDHs, while *Cb*AlaDH displayed the lowest.

**Table 2:** Kinetic values of L-alanine L-AlaDH enzymes with regard to pyruvate. k_cat_, K_M_ and catalytic efficiency (k_cat_/K_M_) are shown.

Enzyme	Pyruvate		
	k_cat_ (s^−1^)	K_M_ (mM)	k_cat_/K_M_ (s^−1^ mM^−1^)
***MetAlaDH***	139.6 ± 19.7	1.3 ± 0.4	108.2 ± 12.0
***AcfhAlaDH***	112.2 ± 6.6	0.7 ± 0.2	148.2 ± 13.5
***VlAlaDH***	62.5 ± 3.4	0.3 ± 0.0	170.0 ± 6.7
***TtAlaDH***	87.0 ± 5.1	0.63 ± 0.2	137.8 ± 15.7
**Cb*AlaDH***	0.5 ± 0.1	0.4 ± 0.0	1.3 ± 0.1
**Af*AlaDH***	15.4 ± 1.0	0.2 ± 0.1	66.7 ± 10

The K_M_ values with regard to α-ketobutyrate varied widely: *Af*AlaDH had the lowest K_M_ value (0.2 mM), while *Vl*AlaDH exhibited the highest value (5.8 mM) (Table 3). Of the L-AlaDHs evaluated in this study, *Af*AlaDH displayed the most favourable catalytic efficiency with regard to α-ketobutyrate, with a value of 34 s^−1^ mM^−1^, while *Cb*AlaDH, an archaeal L-AlaDH, displayed the least favourable catalytic efficiency (0.3 s^−1^ mM^−1^).

**Table 3:** Kinetic values of L-AlaDH enzymes with regard to α-ketobutyrate. k_cat_, K_M_ and catalytic efficiency (k_cat_/K_M_) are shown.

Enzyme	α-ketobutyrate			
	k_cat_ (s^−1^)	K_M_ (mM)	k_cat_/K_M_ (s^−1^ mM^−1^)	
***MetAlaDH***	3.3 ± 0.0	3.8 ± 1.2	0.8 ± 0.0	
***AcfhAlaDH***	5.4 ± 0.5	1.3 ± 0.4	4.0 ± 1.4	
***VlAlaDH***	10.7 ± 0.4	5.8 ± 1.0	1.8 ± 0.2	
***TtAlaDH***	1.1 ± 0.0	0.4 ± 0.1	3.0 ± 0.7	
**Cb*AlaDH***	0.7 ± 0.2	2.2 ± 1.0	0.3 ± 0.2	
**Af*AlaDH***	7.5 ± 0.1	0.2 ± 0.0	34.0 ± 0.1	

The kinetic parameters with regard to α-ketovalerate are shown in (Table 4). As with ketobutyrate, the results showed a wide range of K_M_ values, with the highest value (5.2 mM) observed in *Acfh*AlaDH, and the lowest value observed in *Af*AlaDH (0.3 mM). While *Af*AlaDH displayed the highest catalytic efficiency (2.7 s^−1^ mM^−1^), the lowest catalytic activities were observed in *Cb*AlaDH and *Acfh*AlaDH, with values of 0.21 and 0.2 s^−1^ mM^−1^, respectively.

**Table 4:** Kinetic values of L-AlaDH enzymes with regard to α-ketovalerate. k_cat_, K_M_ and catalytic efficiency (k_cat_/K_M_) are shown.

Enzyme	α-ketovalerate		
	k_cat_ (s^−1^)	K_M_ (mM)	k_cat_/K_M_ (s^−1^ mM^−1^)
***MetAlaDH***	0.7 ± 0.0	1.9 ± 0.0	0.4 ± 0.0
***AcfhAlaDH***	1.0 ± 0.0	5.2 ± 0.8	0.2 ± 0.0
***VlAlaDH***	1.9 ± 0.3	3.3 ± 0.2	0.6 ± 0.0
***TtAlaDH***	1.2 ± 0.0	3.3 ± 1.1	0.4 ± 0.0
**Cb*AlaDH***	0.17 ± 0.1	0.8 ± 0.0	0.21 ± 0.1
**Af*AlaDH***	0.9 ± 0.2	0.3 ± 0.0	2.7 ± 0.3

Michaelis-Menten graphs were created by assaying the L-AlaDH enzymes for each substrate at 25 °C and an optimum pH level to determine the kinetic parameters of the enzyme. GraphPad Prism 9 (GraphPad Software, 2022) was used to determine the kinetic parameters (k_cat_ and K_M_) and the summary of the kinetic parameters obtained for pyruvate (Table 2) revealed significant diversity in both K_M_ and k_cat_ values. The kinetic reactions with pyruvate proceeded reasonably well to determine the kinetic values. All enzymes with α-ketobutyrate showed inaccuracy and also especially with *Cb*AlaDH, *Met*AlaDH and *Tt*AlaDH when α-ketovalerate was the substrate (Figure S2 and Table 3, Table 4). Therefore, these values are used only for indicative comparison. The K_M_ values for the studied enzymes fell within the range 0.2–1.3 mM, while the range of k_cat_ values was 0.5–139.6 s-1 mM-1. Notably, *Met*AlaDH exhibited the highest k_cat_ value among the tested enzymes, and also had the highest K_M_ value. *Vl*AlaDH exhibited the highest catalytic efficiency value of the L-AlaDHs, while *Cb*AlaDH displayed the lowest.

The study found that only *Tt*AlaDH and *Af*ALaDH were active with regard to ketocaproate. Although *Af*AlaDH showed some activity with regard to α-ketocaproate, its kinetic values could not be determined due to difficulties in obtaining values that fitted the Michaelis-Menten model. Only the catalytic efficiency of *Tt*AlaDH was measured, which was 0.6 s^−1^ mM^−1^. However, the *Tt*AlaDH K_M_ value was measured as 0.4 mM, which indicated the good affinity of this bulky substrate for this enzyme. All Michaelis-Menten graphs generated for the various substrates are shown in (Fig. S2).

Table 5 illustrates the conversion efficiency of L-AlaDH, with conversions presented as a percentage change per minute calculated from NADH consumption. The *Acfh*AlaDH enzyme exhibited the highest conversion efficiency, achieving a 57% pyruvate conversion per minute at the given substrate concentration, while *Cb*AlaDH displayed the lowest conversion rate of 7%. The *Vl*AlaDH enzyme demonstrated the highest conversion efficiency with regard to α-ketobutyrate, while *Acfh*AlaDH exhibited the best conversion efficiency with regard to α-ketovalerate. Activity with regard to α-ketocaproate was observed only in *Af*AlaDH and *Tt*AlaDH, with respective conversion efficiencies of 9% and 5%. The obtained results indicated that the L-AlaDH enzymes demonstrate activity with various substrates.

**Table 5:** Conversion efficiency of L-AlaDH enzymes with regard to pyruvate and ketoacids.

Enzyme	Conversion (%)			α-ketocaproate
	Pyruvate	α-ketobutyrate	α-ketovalerate	
***Met*AlaDH**	55	17	12	ND
***Acfh*AlaDH**	57	19	17	ND
***Vl*AlaDH**	54	25	15	ND
***Tt*AlaDH**	49	14	11	5
**CbAlaDH**	7	4	3	ND
**AfAlaDH**	53	17	13	9

### 3.4 Modeling of *Af*AlaDH and *Tt*AlaDH active sites

**Fig. 4:**
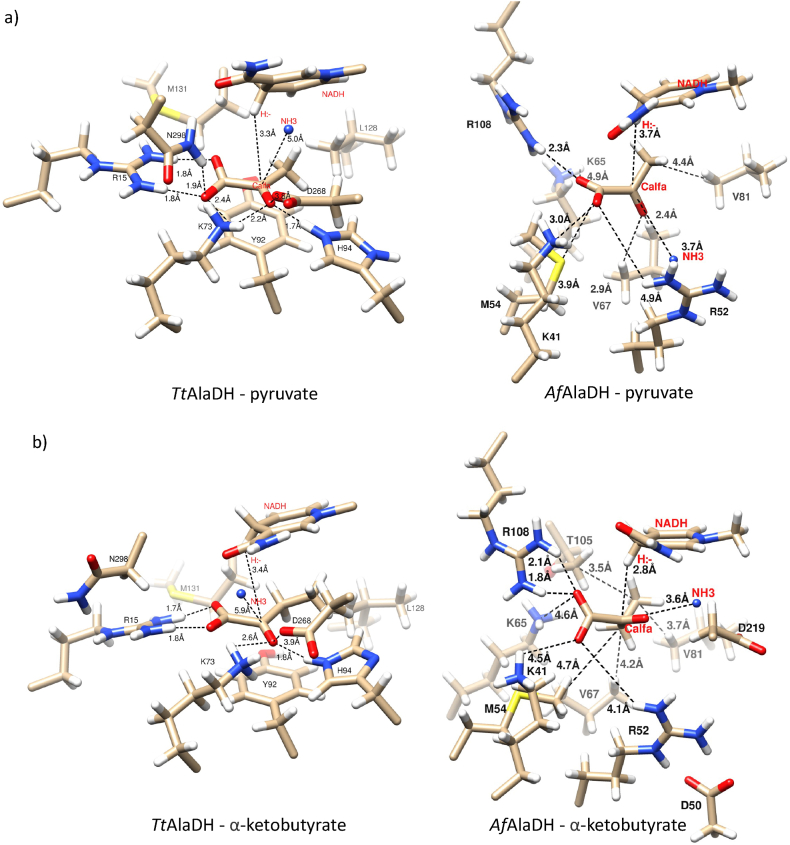

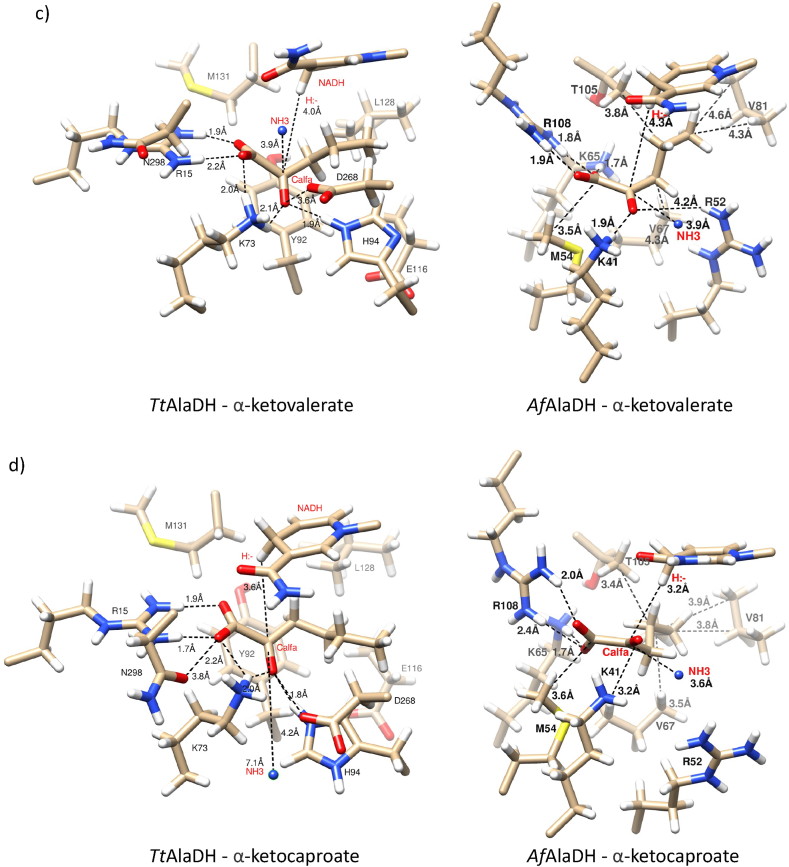
Active site models of l-alanine dehydrogenase (L-AlaDH) from *A. fulgidus* (Af*AlaDH*) and *T. thermophilus* (*TtAlaDH*). The active site structures are shown with key amino acids for the binding of different substrates, NADH and ammonia (NH_3_). a) *TtAlaDH* and Af*AlaDH* with pyruvate and NADH, b) *TtAlaDH* and Af*AlaDH* with α-ketobutyrate and NADH, c) *TtAlaDH* and Af*AlaDH* with α-ketovalerate and NADH, and d) *TtAlaDH* and Af*AlaDH* with α-ketocaproate and NADH. Oxygen is shown in red, nitrogen in blue, carbon in light brown and protons in white. Hydrophilic and hydrophobic interactions are shown between hydrogen and nitrogen or oxygen atoms and between carbon and carbon, respectively. Distances are in Ångströms and comparable values are shown in (Tables S1 and S2). (For interpretation of the references to color in this figure legend, the reader is referred to the Web version of this article.)

There were significant differences between *Af*AlaDH and *Tt*AlaDH enzymes in terms of their amino acid sequences and 3D structures. The multiple sequence alignment and the phylogenetic tree of *AcfhAlaDH,* Af*AlaDH* Cb*AlaDH MetAlaDH*, *TtAlaDH* and *VlAlaDH* were determined, and the alignment and the crystal structures of PDB 1OMO (Af*AlaDH*) and PDB 2EEZ (*TtAlaDH*) are shown in (Fig. S3) and (Fig. S4). Major differences can be explained by the archaeal and bacterial origins of the enzymes. Despite this, the binding interactions were similar at the molecular level. In both enzymes, the spatial orientation of the substrates and NADH binding were comparable (Fig. 4). Notable amino acids in the binding of the substrate (i.e. pyruvate, α-ketobutyrate, α-ketovalerate and α-ketocaproate) were hydrogen-bonding arginine, histidine and lysine.

*There are* in Af*AlaDH hydrophilic contacts of Lys41 and* Arg108 and in *TtAlaDH hydrophilic contacts of* Arg15, Lys73 and also Asn298, which bound strongly (H-bond distance ≤3 Å) to the pyruvate carboxylate group. In addition, the pyruvate carbonyl group can bind *to Lys73, His94 and Asp268* in *TtAlaDH, although* there is no hydrogen binding to the carbonyl group *in* Af*AlaDH*. The distance from ammonia to the active carbon C_alfa_ of pyruvate was closer in Af*AlaDH* (3.7 Å) than in *TtAlaDH* (5.0 Å), although the angle of its entry was from the same direction. The overall reactive orientation was also affected by the hydrophobic interactions. Pyruvate was positioned between two ring structures (Tyr92 and NADH) in *TtAlaDH, while* in Af*AlaDH,* Val67 is present instead of tyrosine. All substrates had a different carbon backbone and that caused constriction when the aliphatic chain was longer than pyruvate. Af*AlaDH and TtAlaDH* have different hydrophobic side chains of amino acids: Af*AlaDH* has smaller amino acid side chains (methionine, threonine and valine) than *TtAlaDH,* although the latter also contains Met131, and also contains Tyr92, His94 and Leu128. Because the distance between hydrogen in the C4 carbon of NADH and the reactive carbonyl group of the substrates is similar, a hydrogen shift may occur with both enzymes. (Fig. 4).

**Fig. 5:**
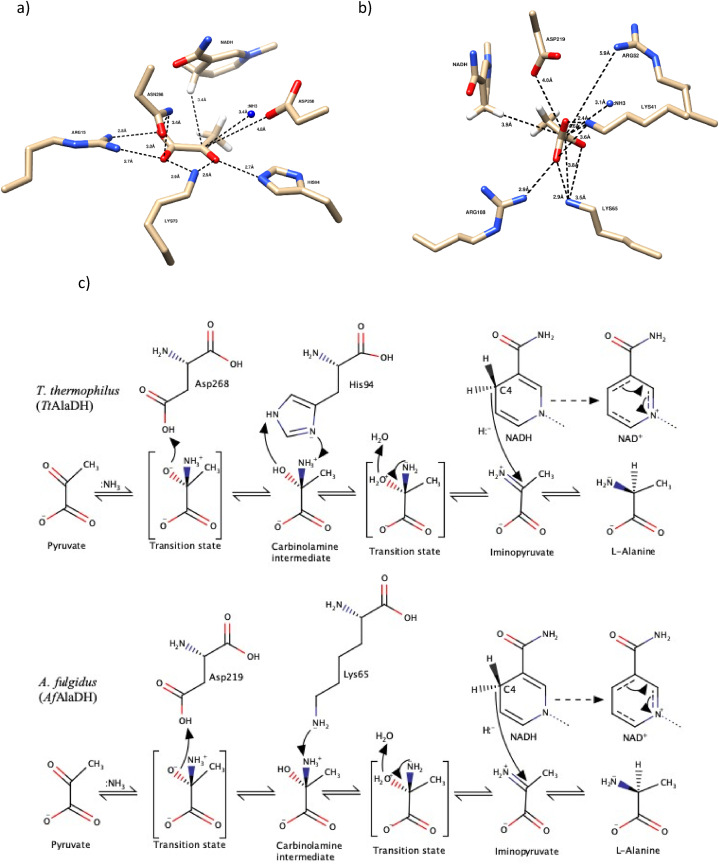
Catalytic mechanism in reverse reaction of *Af*AlaDH and *Tt*AlaDH. The top part of figure shows the catalytic amino acids in the active site of *Tt*AlaDH (a) and *Af*AlaDH (b), ammonium (:NH3) and cofactor NADH, and their distances (Å) from the reactive carboxyl group of pyruvate. c) The catalytic reaction is shown with transition states and intermediate stages.

Fig. 5 provides a detailed depiction of the reductive deamination mechanism of *Af*AlaDH and *Tt*AlaDH.

## 4 Discussion

Interest in the production of UAA is growing due to the increasing number of amino acids and derivatives used in functional food production and in the pharmacochemical industry. Currently, the market value of the amino acid industry is estimated at US$25 billion [39]. As such, enzymes that can be used in the synthesis of UAA have great potential. Different processes for the chemical and enzymatic synthesis of l-amino acids have been reported in the literature, although microbial synthesis has received the most attention as it is cost-efficient and environment-friendly [[15], [16], [17],^40^]. L-AlaDH is an important enzyme in the generation of l-alanine, which is a crucial intermediate in the production of peptidoglycans in microorganisms and plays a critical role in maintaining equilibrium in nitrogen-carbon metabolism [21,41]. Given this feature, efforts have been made to produce various derivatives of l-alanine with different keto acids. However, the yield has remained low, probably due to a low conversion rate. This has attributed to the high selectivity of the L-AlaDH enzyme when pyruvate is used as a substrate [22,23,26,42].

This study evaluated the ability of L-AlaDHs to perform reductive amination with regard to pyruvate and its derivatives (i.e. α-ketobutyrate, α-ketovalerate and α-ketocaproate). There is very limited information in the literature on the reductive deamination reactions of L-AlaDHs. Ammonia plays an important role in the reductive reaction of l-alanine dehydrogenase enzymes where its free electron pair allows it to form bonds with the carbonyl group of the substrate. In the catalytic reaction, ammonia most likely has an effect via nucleophilic substitution (SN1). In order for the amination reaction to occur, ammonia must react with the reactive carbonyl of the substrate, and this step could be assisted by the acidic aspartate in the vicinity of the active site [30,43]. Although the distances between the substrates and the cofactors in our models were average values, they still provide valuable information when comparing the interactions that bind the substrate. Hydrogen bonds to the substrate are mostly formed by charged amino acids, e.g. arginine, histidine and lysine, and these bonds also keep it in place. The models of *Af*AlaDH and *Tt*AlaDH can be used to compare the mechanism of reverse reactions. In *Af*AlaDH, pyruvate is bound by Lys41, Lys65, Arg52 and Arg108, and the reaction is likely to be catalysed by Lys65 and Asp219, since they are in such positions that catalysis occurs. The first step of the reaction is the binding of ammonia to the carbonyl group of pyruvate. Asp219 acts as an acid donor and gives the proton to the first transition state. Lys65 acts then as the base and receives the proton. The carbinolamine intermediate thus formed is positively charged, and an unfavourable repulsion of positive charges is formed. This is eliminated in another transitional state as the water molecule is removed, then iminopyruvate is produced. After that, hydride is transferred from NADH to iminopyruvate, and l-alanine is formed. The corresponding catalytic residues of *Tt*AlaDH are His94 and Asp268, and the residues that are involved in pyruvate binding are Arg15, Lys73 and Asp298. The formation of catalytic intermediates requires conformational changes in the active site. They allow the nucleophilic attack of ammonia on the carbonyl group of the pyruvate, and the removal of the water molecule. In addition, the conformation of the nicotinamide ring of cofactor NADH changes in reaction [28]. Moreover, *Af*AlaDH has a more alkaline optimum pH than *Tt*AlaDH, so the protonation step will be slow at high pH levels [[27], [28], [29], [30], [31]] The *Af*AlaDH and *Tt*AlaDH models are consistent with the observation that lysine is a major acid-base catalyst [31]. In the case with *Af*AlaDH and *Tt*AlaDH, the reverse reaction involves the keto acid substrate, ammonia and NADH.

In the hydride transfer reaction, the active carbonyl group is commonly directed towards the hydrogen of C4 carbon in the NADH ring. The stability of the whole NADH conformation also strongly influences the final phase [29]. As it has been suggested that the final step is a hydride shift in the reduction of the substrate, it is evident that the reaction environment must be anhydrous [27,28]. The aromatic Tyr92 in *Tt*AlaDH (not in *Af*AlaDH) contributes to the maintenance of the active site as a closed conformation and also contributes to the alignment of the carbonyl group of substrates with hydrogen in the NADH ring. The aliphatic side chain of the substrate has interactions with hydrophobic amino acids at the active site. The largest side chain of ketocaproate has the least space at the active site. The most restricting amino acids are Leu128 in *Tt*AlaDH, and Val81 and Thr108 in *Af*AlaDH.

Surprisingly, *Af*AlaDH has Arg52 in the same place as there is His94 in *Tt*AlaDH (His96 in 2 PDB 2VOJ), which is also able to make hydrogen bonds with the carbonyl group of the substrate (Fig. 5). However, as most L-AlaDHs in our study have an optimum pH between 8.5 and 10.5 (with the exception of one L-AlaDH with an optimum at pH 7) (Table 6), it is possible that lysine with a high pKa value could play a larger role in proton shuffling than previously reported. A mutation study that introduced lysine in 25 different internal protein positions reported pKa values between 5.3 and 10.4 [39]. This supports the possibility that lysine has an active role in proton shuffling across a wide pH range. In addition, it has been observed that arginine is predominantly charged inside proteins at pH values as high as 10, due to the high pKa value (13.8) of the guanidium group [40]. This would suggest that, as *Af*AlaDH does not contain histidine and has, lysine close to the carbonyl group, functioning in proton shuffling. Furthermore, Asp219 and Asp268 are most likely to be negatively charged (pKa: 3.8–4.2) but aspartate has catalytic key role in proton movements. Altogether, these considerations should lead to re-evaluation in future studies of the exact role of active site residues in the *Af*AlaDH enzyme [27,31,35] [44,45].

**Table 6:** Optimum pH, temperature and kinetic values for the reported reductive amination reactions of l-alanine dehydrogenase (L-AlaDH) from different species with regard to pyruvate. Optimal pH, k_cat_, K_M_ and temperature optimum values are shown.

Source organism of AlaDH	Expression system	Reductive amination			Temp. (°C)	References
		pH	k_cat_ (s^−1^)	K_M_ (mM)		
*Thermus thermophilus*	*Thermus thermophilus*	8.5	–	0.75	25	[36]
*Phormidium lapideum*	*Phormidium lapideum*	8.4	–	0.33	–	[25]
*Mycobacterium tuberculosis*	*E.coli CAG629*	7.5	–	1.45	–	[47]
*Thermus caldophilus*	*E. coli* *MV1184*	8.0	–	0.20	60	[46]
*Vibrio proteolyticus*	*E.coli* TG1	8.0	–	0.61	50	[24]
*Archaeoglobus fulgidus*	*E.coli* BL21 (DE3)	7.0	118.0	0.16	82	[23]
*Streptomyces anulatus*	*E.coli* BL21 (DE3)	8.5	–	0.80	40	[42]
*Aphanothece halophytica*	*E.coli* BL21 (DE3)	9.0	–	0.22	25	[48]
*Bacillus pseudofirmus*	*E.coli* BL21 (DE3)	10.5	18.0	1.00	40	[49]
*Streptomyces coelicolor*	*E.coli* BL21 (DE3)	9.0	k_cat_/K_M_ (s^−1^ mM) = 1.90		30	[22]
*Helicobacter aurati*	*E.coli* BL21 (DE3)	8.0	–	0.56	55	[26]
*Amycolatopsis sulphurea*	*E.coli* BL21 (DE3)	–	–	–	25	[50]
*Thermomicrobium roseum*	*E.coli* BL21 (DE3)	9.5	35.0	1.9	25	[35]

The optimal temperatures reported in previous studies varies between 25 and 82 °C (Table 6), with most enzymes exhibiting an optimum temperature at or below 50 °C, with the exception of *Af*AlaDH [23] and L-AlaDHs from *Thermus caldophilus* [46], and *Helicobacter aurati* [26]. In our study, we found that *Met*AlaDH, *Vl*AlaDH and *Acfh*AlaDH have an optimum temperature at 50 °C. The archaeal enzyme *Cb*AlaDH was the most thermotolerant in our study with a temperature optimum of 65 °C and demonstrated significant activity even at 80–90 °C (Fig. 3). Notably, this optimum value was found to be lower than the temperature optimum of *Af*AlaDH, the sole archaeal L-AlaDH previously characterized in the literature, which exhibited maximal activity at 82 °C [23].

As shown in Table 6, the optimal pH range for the enzymes examined in previous studies is mostly alkaline (pH 7.0–10.5). The enzymes in our study showed a similar pH range (7.0–10.5). The average pH optimum was between 8.0 and 9.5 and only a few enzymes have an optimal value at pH 7.0 (Table 1, Table 6). These findings showed that the alkaline nature of L-AlaDHs is a common feature.

All L-AlaDHs showed activity with regard to pyruvate, α-ketobutyrate and α-ketovalerate (Table 1). *Met*AlaDH had the highest specific activity with regard to pyruvate among all studied enzymes. All showed greater activity with regard to pyruvate than other substrates. Among the L-AlaDHs, the most preferred substrate after pyruvate was α-ketobutyrate. Our results showed that L-AlaDHs were able to catalyse the reaction for α-ketobutyrate and α-ketovalerate (Table 1), although they did not exhibit significant activity towards α-ketocaproate, which may be due to the increased size of the aliphatic sidechain. Only *Tt*AlaDH and *Af*AlaDH showed detectable activity with α-ketocaproate. Based on current understanding [51], it is apparent that α-ketocaproate cannot position itself properly or orient itself within the active site of L-AlaDHs to undergo catalytic conversion. Our examination revealed that the K_M_ values for pyruvate (Table 2) ranged from 0.2 to 1.3. Our results showed that the K_M_ value for *Met*AlaDH was higher than that of most studied L-AlaDH values in previous studies (Table 6). Moreover, our research revealed that the K_M_ values for pyruvate, which ranged between 0.2 and 1.3, are similar as found in previous studies (Table 2, Table 6). The k_cat_ values for pyruvate also varied widely (0.5–139.6) (Table 2). It should be noted that the k_cat_ value of *Met*AlaDH (Table 2) is higher than the values reported in previous studies (Table 6)**.** In our study, *Vl*AlaDH showed the best catalytic efficiency (170 s^−1^ mM^−1^) with regard to pyruvate followed by *Acfh*AlaDH (148.2 s^−1^ mM^−1^). Since the K_M_ value did not vary as much as k_cat_ between the different enzymes, it is evident that the variation in the catalytic efficiency was caused by considerable variation in the catalytic rate of the enzyme reaction.

Among all studied enzymes, *Vl*AlaDH exhibited the highest specific activity towards α-ketobutyrate and α-ketovalerate at 13.2 U/mg and 1.9 U/mg, respectively (Table 1). This is in line with the higher catalytic efficiency of *Af*AlaDH towards α-ketobutyrate and α-ketovalerate, with values of 34.0 s^−1^ mM^−1^ and 2.7 3 s^−1^ mM^−1^, respectively (Table 3, Table 4). While most enzymes did not react with α-ketocaproate, probably due the large size of the molecule, the *Af*ALaDH enzyme exhibited the highest specific activity towards α-ketocaproate (2.8 ± 0.3 U/mg) of the two enzymes that accepted α-ketocaproate as a substrate (Table 1). However, due to an inability to obtain values that align with the Michaelis-Menten model, K_M_ and k_cat_ could not be calculated. Only *Tt*AlaDH showed measurable K_M_ and k_cat_ values with regard to α-ketocaproate, with values of 0.4 mM and 0.6 s^−1^ mM^−1^, respectively. The diversity of substrates accepted by the studied L-AlaDHs indicates that it might be possible to develop new enzymes in this family that can be used to efficiently building green/sustainable pharmaceutically useful UAA.

The existing studies on the synthesis of various alanine derivatives using different keto acids includes only limited amount of reports on K_M_ and k_cat_ values [[21], [22], [23],^26^,^41^]. Thus, our study sought to evaluate the potential of newly identified L-AlaDHs and previously studied L-AlaDHs (from the literature) to synthesise alanine derivatives, with a particular focus on determining their corresponding kinetic values. The values presented in Table 5 indicate promising reaction rates of the enzyme with the substrates used in the direction of reductive amination. The calculations pertain to conversion rates at low substrate concentrations. However, it is imperative to conduct more comprehensive studies on the conversion rates of L-AlaDH toward various substrates.

The present study provides valuable insights into the potential applications of L-AlaDH in various biotechnological domains, such as the development of antimicrobial drugs and biosensors. Furthermore, the outcomes of this research indicate that *Vl*AlaDH, *Met*AlaDH and *Acfh*AlaDH, also *Af*AlaDH and *Tt*AlaDH from earlier studies, have the potential to act as suitable systems in future studies that focus on protein engineering aimed at improving the functional and catalytic properties of L-AlaDH for various biotechnological applications.

## 5 Conclusions

The primary aim of this study was to investigate the properties and functionality of L-AlaDH enzymes with regard to their ability to catalyse the reductive amination of pyruvate and its derivatives. To achieve this goal, the enzymes were expressed through a heterologous system in *E. coli* BL21 (DE3) cells and subsequently purified to obtain a homogenous sample for analysis. The results indicated that L-AlaDH enzymes have considerable potential to use pyruvate and its keto acid derivatives as substrates in reductive amination reactions. These enzymes were found to be effective in the synthesis of optically active unnatural chiral amino acids, which have important applications in various fields, such as pharmaceuticals, agriculture, and food industries. The ability of L-AlaDH enzymes to catalyse the reductive amination of pyruvate and its derivatives could provide a sustainable and cost-effective alternative to conventional chemical methods for the synthesis of these valuable compounds.

Enzyme engineering methods can play a crucial role in the development of L-AlaDH enzymes for different applications. By using various techniques, such as rational design, directed evolution and computational approaches, it is possible to modify the active site of the enzyme to enhance its catalytic activity, substrate specificity and affinity for relevant substrates. These modified enzymes can have various applications, including biocatalysis, drug discovery and biotechnology. Furthermore, understanding the structure-function relationship of L-AlaDH enzymes can aid in the development of novel enzymes with improved characteristics, making them useful tools in various industrial applications.

## Data availability statement

Data included in article/supp. material/referenced in article.

## CRediT authorship contribution statement

**Ğarip Demir:** Writing – review & editing, Writing – original draft. **Jarkko Valjakka:** Writing – review & editing. **Ossi Turunen:** Writing – review & editing. **Fatih Aktaş:** Data curation. **Barış Binay:** Writing – review & editing.

## Declaration of competing interest

The authors declare the following financial interests/personal relationships which may be considered as potential competing interestsBaris Binay reports financial support was provided by The 10.13039/501100004410Scientific and Technological Research Council of Turkey (TUBITAK). Baris Binay reports a relationship with The 10.13039/501100004410Scientific and Technological Research Council of Turkey that includes: funding grants. This work was supported partially by The 10.13039/501100004410Scientific and Technological Research Council of Turkey (TUBITAK) (Project number: 120Z501). If there are other authors, they declare that they have no known competing financial interests or personal relationships that could have appeared to influence the work reported in this paper.
