# Supplementary material for: Cloning and expression heterologous alanine dehydrogenase genes: Investigation of reductive amination potential of L-alanine dehydrogenases for green synthesis of alanine derivatives
Source: Heliyon. 2024 Feb 29;10(5):e26899. doi: 10.1016/j.heliyon.2024.e26899 (PMC10923667; doi:10.1016/j.heliyon.2024.e26899)
Supplement: Multimedia component 1 [file mmc1.docx]

Supplementary information

**Cloning and expression heterologous alanine dehydrogenase genes: Investigation reductive amination potential of L-alanine dehydrogenases for green synthesis of alanine derivatives.**

Ğarip Demir^a^, Jarkko Valjakka^b^, Ossi Turunen^c^ , Fatih Aktaş ^d^, and Barış Binay^e,f*^

^a^ Department of Molecular Biology and Genetics, Gebze Technical University, 41400, Gebze, Kocaeli, Turkey

^b^ Faculty of Medicine and Health Technology, Tampere University, FI-33100 Tampere, Finland

^c^ School of Forest Sciences, University of Eastern Finland, FI-80101 Joensuu, Finland

^d^ Faculty of Engineering, Düzce University, 81600 Düzce, Turkey

^e^ Department of Bioengineering, Gebze Technical University, 41400, Gebze, Kocaeli, Turkey

^f^ BAUZYME Biotechnology Co., Gebze Technical University Technopark, 41400, Gebze, Kocaeli, Turkey

***Corresponding author:** **Assoc. Prof. Dr. Barış Binay,**

Department of Bioengineering, Gebze Technical University,

Gebze, Kocaeli, Turkey, Tel: +90 262 605 22 80

E-mail: [binay@gtu.edu.tr](mailto:binay@gtu.edu.tr)


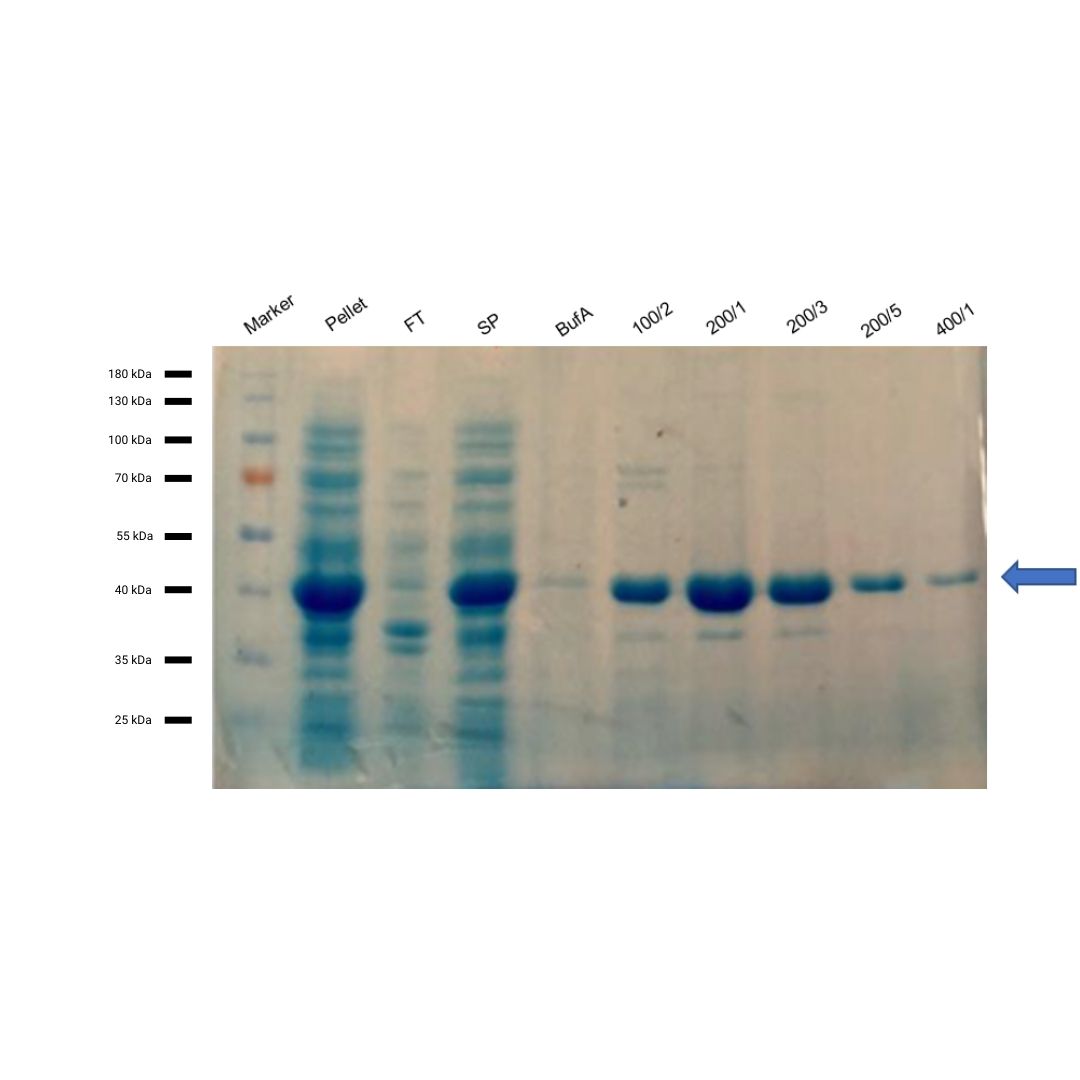


**Figure S1 a)**. SDS-PAGE gel analysis after *Melghiribacillus thermohalophilus* alanine dehydrogenase (*Met*AlaDH) purification. Lanes: Marker, Pellet, FT (solution eluted from the column), SP (supernatant), Buffer A (BufA), and elution fractions with 100 mM, 200 mM and 400 mM imidazole. In addition, Buffer A consisted of 20 mM NaPi, 500 mM NaCl and 30 mM [imidazole](https://www.sciencedirect.com/topics/chemical-engineering/imidazole) at pH 7.4. During the protein collection process, three separate 1 ml fractions were obtained in 100 mM imidazole, five 1 ml fractions in 200 mM imidazole, and three 1 ml fractions in 400 mM imidazole.


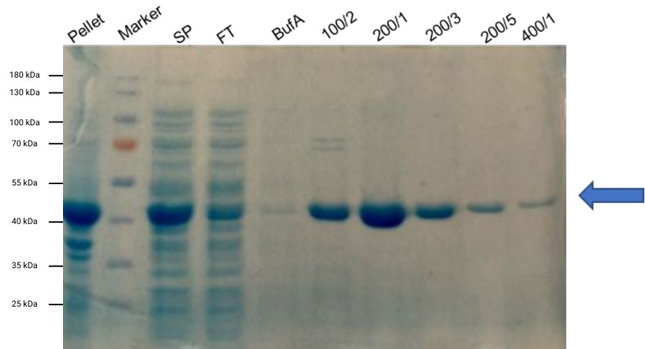


**Figure S1 b)**. SDS-PAGE gel analysis after *Ammoniphilus sp.* CFH 90114 alanine dehydrogenase (*Acfh*AlaDH) purification. Lanes: Pellet, Marker, SP (supernatant), FT (solution eluted from the column), Buffer A (BufA), and elution fractions with 100 mM, 200 mM and 400 mM imidazole. Buffer A as in Figure S1a. During the protein collection process, three separate 1 ml fractions were obtained in 100 mM imidazole, five 1 ml fractions in 200 mM imidazole, and three 1 ml fractions in 400 mM imidazole.


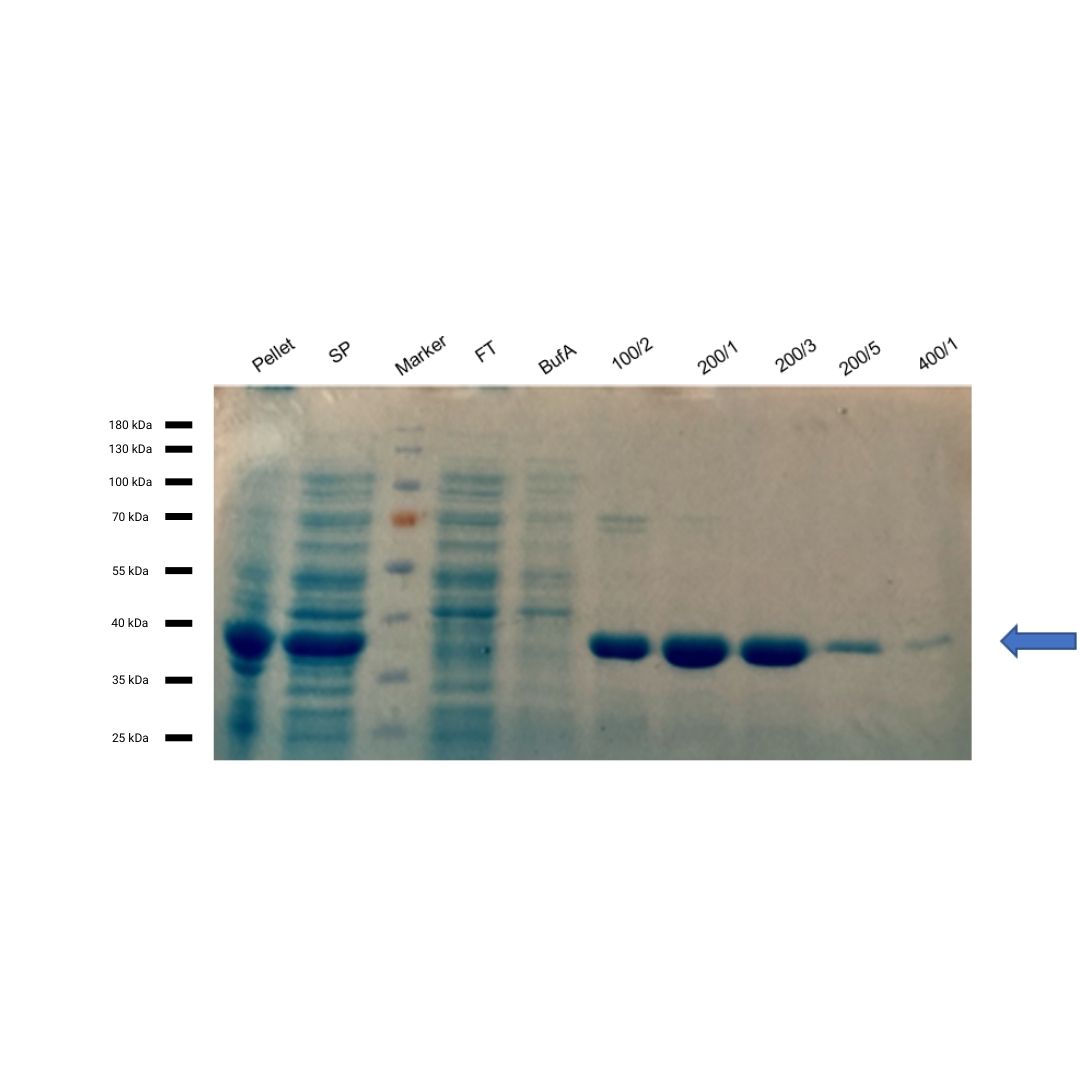


**Figure S1 c)**. SDS-PAGE gel analysis after *Candidatus Bathyarchaeota archaeon* alanine dehydrogenase (*Cb*AlaDH) purification. Lanes: Pellet, SP (supernatant), Marker, FT (solution eluted from the column), Buffer A (BufA), and elution fractions with 100 mM, 200 mM and 400 mM imidazole. Buffer A as in Figure S1a. During the protein collection process, three separate 1 ml fractions were obtained in 100 mM imidazole, five 1 ml fractions in 200 mM imidazole, and three 1 ml fractions in 400 mM imidazole.


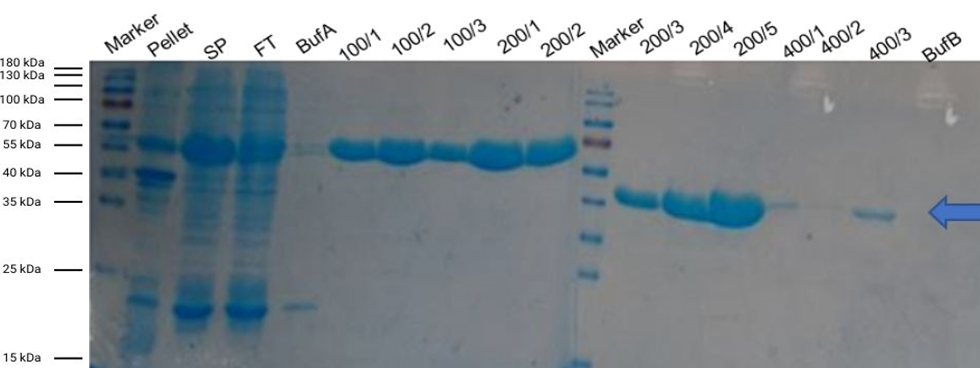


**Figure S1 d)**. SDS-PAGE gel analysis after *Vagococcus lutrae* alanine dehydrogenase (*Vl*AlaDH) purification. Lanes: Marker, Pellet, SP (supernatant), FT (solution eluted from the column), Buffer A (BufA), and elution fractions with 100 mM and 200 mM mM imidazole, Marker, Buffer B (BufB) elution fractions with 200 mM and 400 mM imidazole, and Buffer B. Buffer A, as in Figure S1a. Buffer B contained 20 mM NaPi, 500 mM NaCl and 500 mM imidazole at pH 7.4. During the protein collection process, three separate 1 ml fractions were obtained in 100 mM imidazole, five 1 ml fractions in 200 mM imidazole, and three 1 ml fractions in 400 mM imidazole in Buffer A. In addition, a 5 ml fraction was collected with elution by 500 mM imidazole in Buffer B (BufB lane).


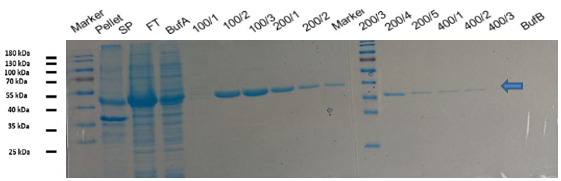


**Figure S1 e)**. SDS-PAGE gel analysis after *Archaeoglobus fulgidus* alanine dehydrogenase (*Af*AlaDH) purification. Lanes: Marker, Pellet, SP (supernatant), FT (solution eluted from the column), Buffer A (BufA), and elution samples with 100 mM and 200 mM imidazole and 400 mM imidazole, Buffer A, as in Figure S1a. During the protein collection process, three separate 1 ml fractions were obtained in 100 mM imidazole, five 1 ml fractions in 200 mM imidazole, and three 1 ml fractions in 400 mM imidazole.


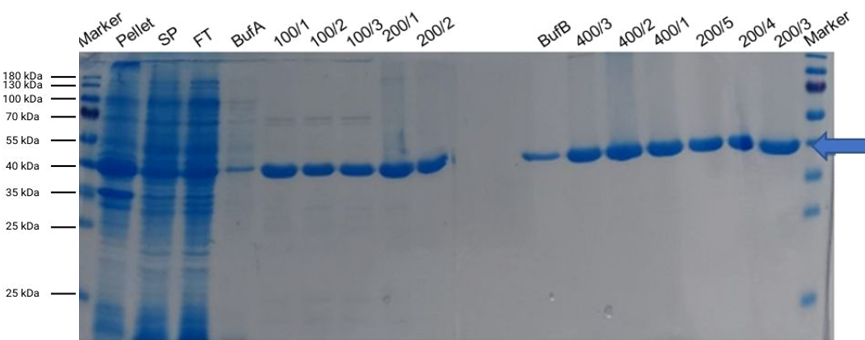


**Figure S1 f)**. SDS-PAGE gel analysis after *Thermus thermophilus* alanine dehydrogenase (*Tt*AlaDH) purification. Lanes: Marker, Pellet, SP (supernatant), FT (solution eluted from the column), Buffer A (BufA), elution fractions with 100 mM and 200 mM imidazole, Buffer B (BufB), elution fractions with 400 and 200 mM imidazole, and Marker. Buffer A, as in Figure S1a. Buffer B contained 20 mM NaPi, 500 mM NaCl, and 500 mM imidazole at pH 7.4. During the protein collection process, three separate 1 ml fractions were obtained in 100 mM imidazole, five 1 ml fractions in 200 mM imidazole, and three 1 ml fractions in 400 mM imidazole in Buffer A. In addition, a 5 ml fraction was collected with elution by 500 mM imidazole in Buffer B (BufB lane).

A

**Figure S2 a)**. Michaelis-Menten graphs of AlaDH enzymes with pyruvate as substrate.

**Figure S2 b)**. Michaelis-Menten graphs of AlaDH enzymes with α-ketobutyrate as substrate.

**Figure S2 c)**. Michaelis-Menten graphs of AlaDH enzymes with α-ketovalerate as substrate.

**Figure S2 d)**. Michaelis-Menten graph of *Tf*AlaDH with α-ketocaproate as substrate.


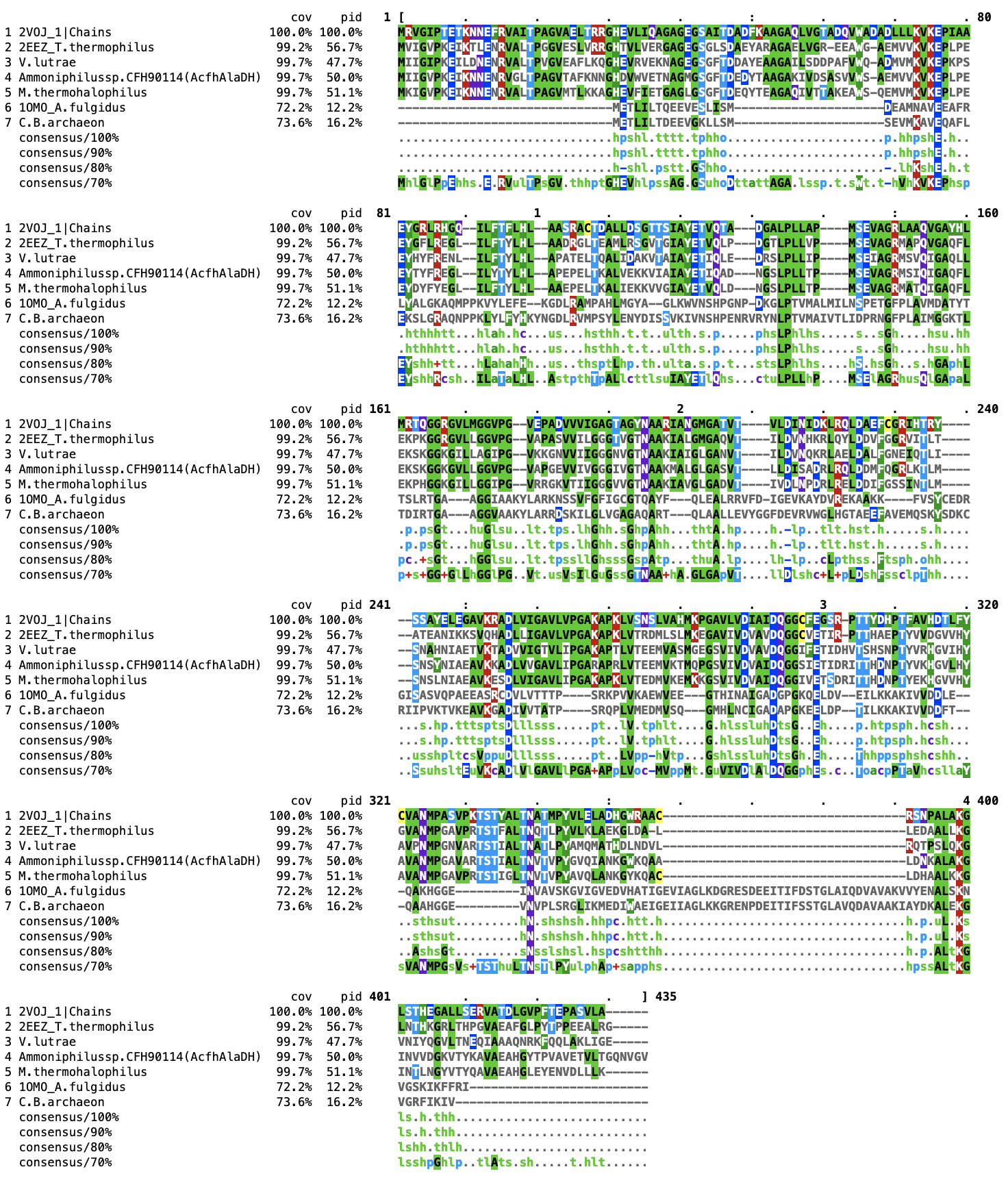


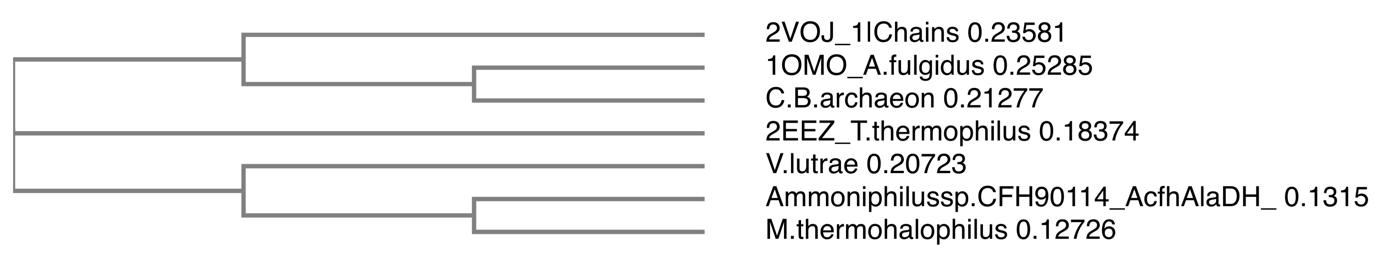


**Figure S3**. Multiple sequence alignment of L-alanine dehydrogenases and their phylogenetic tree. There are noticeable differences in the order and number of amino acids. Sequences were compared to *M. tuberculosis* AlaDH (2voj.pdb).


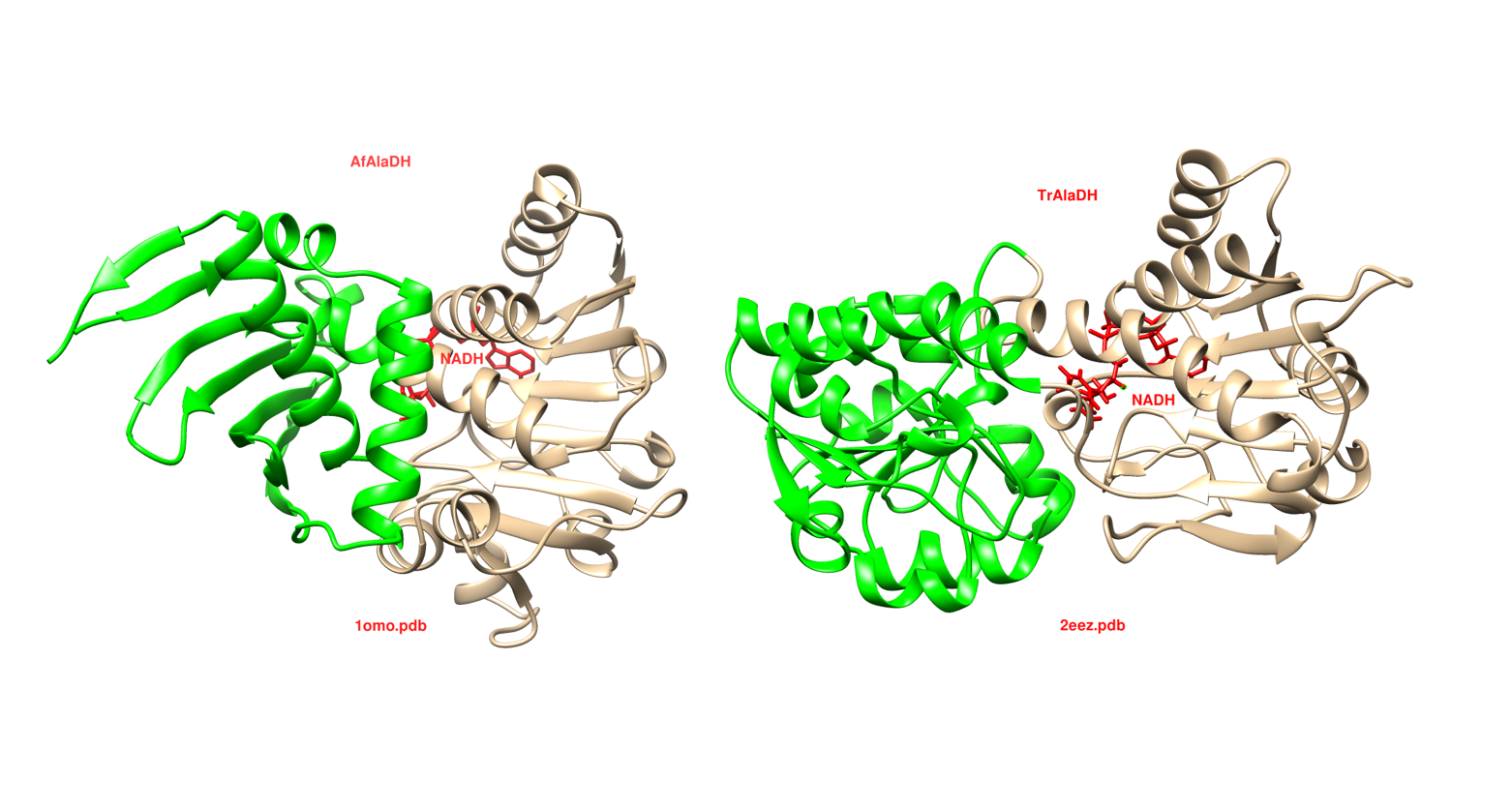


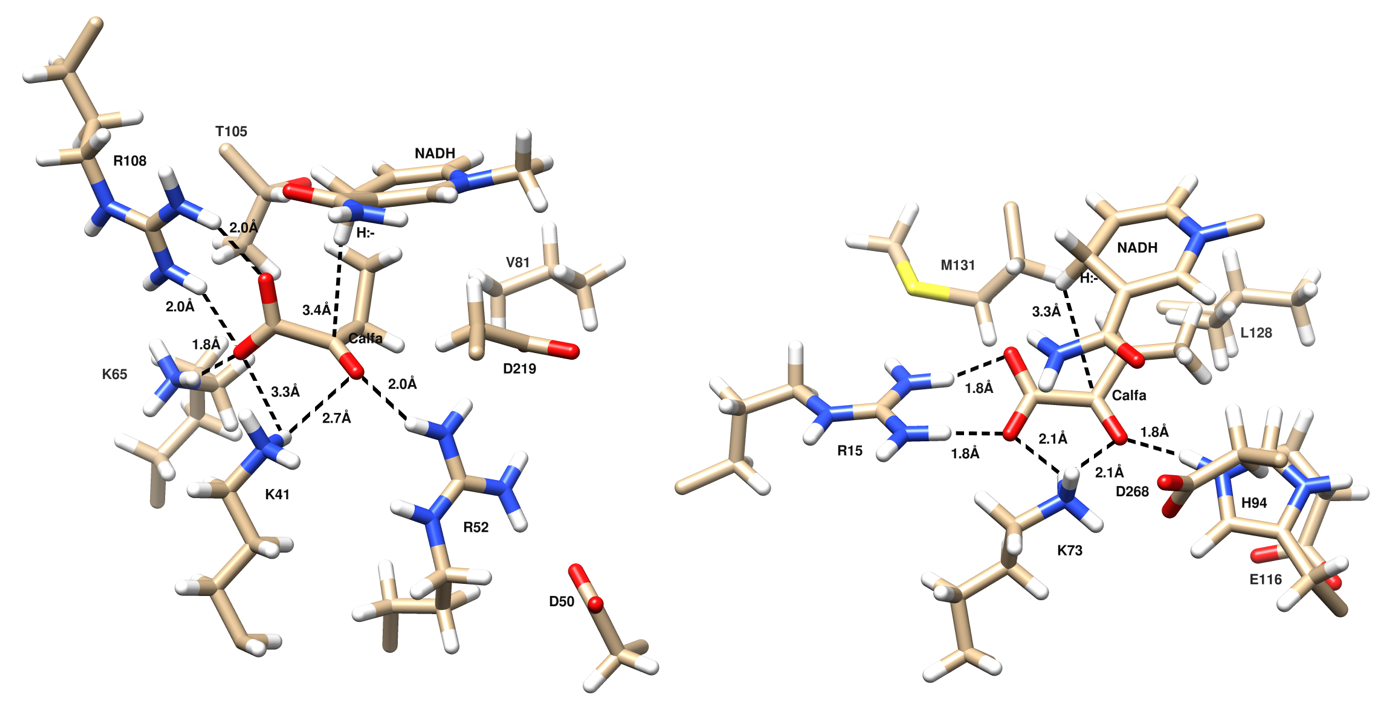


**Figure S4**. The structures of *Af*AlaDH and *Tr*AlaDH enzymes. Upper: The most significant difference between the enzymes is the main folding of the substrate binding structures (green). The NADH (red) binding structures (tan) are highly conserved in all dehydrogenases. Lower: Common hydrogen bond contacts are shown between the substrate and NADH.

| **Table S1**. Summary of the distances from protein modeling of alanine dehydrogenase from *Thermus thermophilus* (PDB code 2EEZ). The table includes the distances of hydrophilic and hydrophobic interactions measured between the amino acids in the active site and pyruvate, ketobutyrate, ketovalerate and ketocaproate, as well as the cofactor NADH. The distance limit is about 5 Å. In addition, the distance of the hydride (H:^-^) and its approach angle to the nucleophilic carbon is shown. | | | | | | | | | |
| --- | --- | --- | --- | --- | --- | --- | --- | --- | --- |
| 2EEZ with NADH | | Hydrophilic | | | | | | | |
|  |  | Pyruvate | | α-ketobutyrate | | α-ketovalerate | | α-ketocaproate | |
|  | Arg15 | 1.8/1.8 | | 1.7/1.8 | | 1.9/2.2 | | 1.7/1.9 | |
|  | Lys73 | 2.2/2.4 | | 2.6 | | 2.0/2.1 | | 2.0/2.2 | |
|  | His94 | 1.7 | | 1.8 | | 1.9 | | 1.8 | |
|  | Asp268 | 3.8 | | 3.9 | | 3.6 | | 4.2 | |
|  | Asn298 | 1.9 | | 5.9 | | out | | 3.8 | |
| Distance NH_3_ and C=O | | 5.0 | | 5.9 | | 3.9 | | 7.1 | |
| Angle NH_3_ and C=O | | 63.0 | | 65 | | 103 | | 29 | |
| Distance H:^-^ and C=O | | 3.3 | | 3.4 | | 4.0 | | 3.6 | |
| Angle H:^-^ and C=O | | 138 | | 145 | | 165 | | 71 | |
| 2EEZ with NADH | | | Hydrophobic | | | | | | |
|  |  |  | Pyruvate | | α-ketobutyrate | | α-ketovalerate | | α-ketocaproate |
|  | Tyr92 | | 3.5 | | 3.5 | | 3.6 | | 3.5 |
|  | His94 | | 4.2 | | 3.6 | | 3.6 | | 3.4 |
|  | Glu116 | | 5.2 | | 5.0 | | 5.6 | | 4.8 |
|  | Leu128 | | 3.7 | | 3.4 | | 3.8 | | 3.9 |
|  | Met131 | | 4.9 | | 4.0 | | 5.5 | | 5.3 |
|  | Asp268 | | out | | 5.7 | | 4.0 | | 3.7 |

| **Table S2**. Summary of distances from protein modeling of alanine dehydrogenase from *Archaeoglobus fulgidus* (PDB code 1OMO). The table includes the distances of hydrophilic and hydrophobic interactions measured between the amino acids of the active site and pyruvate, ketobutyrate, ketovalerate and ketocaproate, as well as the cofactor NADH. The distance limit is about 5 Å. In addition, the distance of the hydride (H:^-^) and its approach angle to the nucleophilic carbon are shown. | | | | | |
| --- | --- | --- | --- | --- | --- |
| 1OMO with NADH | | Hydrophilic | | | |
|  |  | Pyruvate | α-ketobutyrate | α-ketovalerate | α-ketocaproate |
|  | Lys41 | 3.00 | 4.5 | 1.9 | 3.2 |
|  | Arg52 | 4.9 | 4.1 | 4.2 | out |
|  | Lys65 | 4.9 | 4.6 | 1.7 | 1.7 |
|  | Arg108 | 2.3 | 1.8/2.1 | 1.8/1.9 | 2.0/2.4 |
| Distance NH_3_ and C=O | | 3.7 | 3.6 | 3.9 | 3.6 |
| Angle NH_3_ and C=O | | 25.9 | 36 | 30 | 65 |
| Distance H:^-^ and C=O | | 3.7 | 2.8 | 4.3 | 3.2 |
| Angle H:^-^ and C=O | | 153 | 72.6 | 109 | 128 |
| 1OMO with NADH | | Hydrophobic | | | |
|  |  | Pyruvate | α-ketobutyrate | α-ketovalerate | α-ketocaproate |
|  | Thr105 | 3.6 | 3.5 | 3.8/3.9 | 3.4 |
|  | Met54 | 3.9 | 4.7 | 3.5 | 3.6 |
|  | Val67 | 2.4/2.9 | 4.2 | 4.3 | 3.5 |
|  | Val81 | 4.4 | 3.7 | 4.3/4.6 | 3.8/3.9 |
